# DIANA-LncBase v3: indexing experimentally supported miRNA targets on non-coding transcripts

**DOI:** 10.1093/nar/gkz1036

**Published:** 2019-11-16

**Authors:** Dimitra Karagkouni, Maria D Paraskevopoulou, Spyros Tastsoglou, Giorgos Skoufos, Anna Karavangeli, Vasilis Pierros, Elissavet Zacharopoulou, Artemis G Hatzigeorgiou

**Affiliations:** 1 DIANA-Lab, Department of Electrical and Computer Engineering, Univ. of Thessaly, 38221 Volos, Greece; 2 Hellenic Pasteur Institute, 11521 Athens, Greece; 3 Department of Computer Science and Biomedical Informatics, Univ. of Thessaly, 351 31 Lamia, Greece; 4 Department of Informatics and Telecommunications, Postgraduate Program: ‘Information Technologies in Medicine and Biology’, University of Athens, 15784 Athens, Greece

## Abstract

DIANA-LncBase v3.0 (www.microrna.gr/LncBase) is a reference repository with experimentally supported miRNA targets on non-coding transcripts. Its third version provides approximately half a million entries, corresponding to ∼240 000 unique tissue and cell type specific miRNA–lncRNA pairs. This compilation of interactions is derived from the manual curation of publications and the analysis of >300 high-throughput datasets. miRNA targets are supported by 14 experimental methodologies, applied to 243 distinct cell types and tissues in human and mouse. The largest part of the database is highly confident, AGO-CLIP-derived miRNA-binding events. LncBase v3.0 is the first relevant database to employ a robust CLIP-Seq-guided algorithm, microCLIP framework, to analyze 236 AGO-CLIP-Seq libraries and catalogue ∼370 000 miRNA binding events. The database was redesigned from the ground up, providing new functionalities. Known short variant information, on >67,000 experimentally supported target sites and lncRNA expression profiles in different cellular compartments are catered to users. Interactive visualization plots, portraying correlations of miRNA–lncRNA pairs, as well as lncRNA expression profiles in a wide range of cell types and tissues, are presented for the first time through a dedicated page. LncBase v3.0 constitutes a valuable asset for ncRNA research, providing new insights to the understanding of the still widely unexplored lncRNA functions.

## INTRODUCTION

Large scale analyses have turned non-coding RNAs (ncRNAs) into a research hotspot. ncRNAs, such as microRNAs (miRNAs) and long non-coding RNAs (lncRNAs), are being extensively researched for their crucial implication in a remarkable variety of physiological and pathological states (1).

miRNAs are short ncRNAs that act as central post-transcriptional regulators of gene expression (2). They are loaded into protein Argonaute (AGO) to induce target cleavage, degradation and/or translational suppression (3). LncRNAs are typically >200 nucleotides long. They are relatively abundant molecules of the mammalian transcriptome, yet consistently expressed at lower levels compared to protein coding transcripts (4). In contrast to earlier bulk RNA-Seq analyses, recent studies performed at single cell level reveal the high expression of numerous lncRNAs in individual cells (5). The widely studied variability in lncRNA subcellular localization may pinpoint their core action (6). Generally, they are involved in every known level of gene regulation including protein synthesis, RNA maturation, RNA transport and gene silencing (6), while several studies demonstrate that even in low abundances, lncRNAs may serve as prognostic indicators in different pathological states (7).

Recent studies interrogate and validate miRNA–lncRNA interactions in both the nuclear and cytoplasmic compartments, including direct miRNA targeting of nuclear-retained lncRNAs. For instance, miR-101 and miR-217 regulate the half-life of metastasis associated lung adenocarcinoma transcript 1 (MALAT1) in carcinoma cells (8), while miR-449a silences the nuclear enriched abundant transcript 1 (NEAT1) in lung cancer (9). LncRNA may also act as ‘sponge’ for miRNAs, an activity known as ‘endogenous competing RNA’ (ceRNA), reducing the suppressive miRNA-effect on target–mRNAs. In particular, intergenic-muscle differentiation 1 lncRNA (linc-MD1) plays a crucial role in myogenesis by sequestering both miR-133 and miR-135 (10), while lncRNA H19 mediates muscle differentiation by acting as a sponge for let-7 miRNA (11).

miRNA–lncRNA interplay increases the complexity of the multifaceted post-transcriptional gene regulation. Meticulous cataloguing of these interactions constitutes the backbone for future studies aiming to understand their functional consequences.

### Experimental methodologies

Numerous experimental techniques have emerged aiming to delineate miRNA–target pairs in a specific or wider scale (12). Specifically for miRNA–lncRNA interactions, low-yield techniques such as reporter gene assays focus on the identification of the exact miRNA binding location, while qPCR and northern blotting address the regulation of lncRNAs by miRNAs via quantifying change in their abundance. Specific methods may also be applied to indirectly define lncRNA ‘sponges’ by evaluating the silencing mechanism of miRNAs on target–mRNAs, in altered lncRNA concentration levels. High-throughput methodologies, such as microarrays, following miRNA overexpression/knockdown, are considered the extension of specific techniques, enabling the indirect characterization of numerous miRNA–target pairs.

Crosslinking and immunoprecipitation experiments followed by sequencing methodologies (CLIP-Seq) enable the identification of cell type and tissue specific miRNA–target pairs on a transcriptome-wide scale. AGO HITS-CLIP (high-throughput sequencing of RNA isolated by crosslinking immunoprecipitation) (13), PAR-CLIP (photoactivatable-ribonucleoside-enhanced crosslinking and immunoprecipitation) (14), CLEAR-CLIP (covalent ligation and endogenous Argonaute-bound RNA) (15) and CLASH (crosslinking, ligation and sequencing of hybrids) (16) experiments are considered the avant-garde methodologies for direct detection of numerous miRNA binding sites on coding and non-coding transcripts. The latter two techniques are followed by an extra ligation step to acquire chimeric miRNA–target fragments.

### Databases indexing miRNA–lncRNA interactions

Over the past decade, numerous databases have been cataloguing miRNA–mRNA targets. miRNA–lncRNA pairs have not yet been comprehensively defined.

StarBase v2 (17) indexes a compilation of RNA binding events, derived from analysis of numerous CLIP-Seq data and currently hosts ∼36 000 miRNA-ncRNA interactions. The database intersects AGO-enriched regions with *in silico* predicted sites on lncRNA transcripts, derived from miRanda algorithm (18). NPInter v3 (19) provides a collection of experimentally supported ncRNA targets. It includes >70 000 miRNA–lncRNA entries, retrieved either from manual curation of publications or by intersecting computationally predicted sites with AGO-CLIP-Seq data. LncReg (20) substantially differs in its scope by primarily providing lncRNA-associated regulatory entries. It hosts a small number of miRNA–lncRNA targets from low-yield experiments. miRsponge (21) and LncACTdb v2 (22) databases provide lncRNAs with a sponge function. The former hosts ∼600 miRNA–sponge interactions, while the latter provides ∼2663 experimentally supported ceRNA relationships.

In this publication, we present DIANA-LncBase v3.0, an extensive repository with ∼240 000 experimentally supported tissue and cell type specific miRNA–lncRNA interactions in human and mouse species. This compilation of targets, supported by distinct methodologies and experimental conditions, corresponds to half a million miRNA–lncRNA entries. The extensive collection of interactions has been derived from the manual curation of publications and the analysis of more than 300 high-throughput datasets.

Notably, LncBase v3.0 is the first relevant database that employs an advanced CLIP-Seq analysis framework, aiming to catalogue highly confident miRNA-binding events. 236 AGO-CLIP-Seq libraries have been re-analyzed with microCLIP (23), an algorithm specifically deployed for the CLIP-Seq-guided detection of miRNA interactions. The new database version also catalogues (i) direct miRNA–lncRNA chimeric fragments, derived from CLEAR-CLIP and a previous meta-analysis of published AGO-CLIP-Seq datasets (24) and (ii) miRNA–lncRNA pairs, retrieved from the analysis of microarray miRNA perturbation experiments. 86 microarray libraries followed by miRNA-specific transfection/knockdown have been analyzed with an in-house developed pipeline to incorporate hundreds of miRNA–lncRNA interactions into the repository. This collection of high-throughput datasets corresponds to a 2-fold increase compared to LncBase v2.0 (25).

Known short variant information on 67 966 miRNA target sites, from the reference databases dbSNP (26), ClinVar (27) and COSMIC (28), is a new feature in LncBase v3.0. Particular attention has been paid to assemble lncRNA transcript expression profiles in a wide range of cell types/tissues and different cellular compartments (nucleus/cytoplasm), for both human and mouse species, by analyzing 103 raw RNA-Seq libraries, encompassing ∼19.3 billion reads.

The new interface was redesigned from the ground up to facilitate user navigation through the database content by applying different filtering combinations of cell types, tissues and methodologies without performing any specific query. A separate page has been designed to summarize the abundance of lncRNAs in different cell types, as well as their subcellular localization, in nucleus and/or cytoplasm.

Interactive visualization plots portraying (i) the possible clustering of miRNA–lncRNA interactions in different cell types/tissues and (ii) the expression profiles of lncRNAs, are provided through a dedicated results page. A concise description of LncBase v3.0 is presented in Table 1.

**Table 1.** The table summarizes the LncBase v3.0 content in comparison with the experimental module of the previous database version

		LncBase v3.0	LncBase v2.0
Database	Total miRNA–lncRNA entries	>500 000	∼170 000
	Interactions from low-yield methods	242	86
	Interactions from high-throughput methods	∼239 000	∼70 000
	miRNAs in interactions	1551	1419
	Targeted LncRNAs	24 618	8216
	Cell types	192	57
	Tissues	51	22
	Publications	236	59
	lncRNA Resources	GENCODE v30, RefSeq 109–106, Cabili *et al.*	GENCODE v21, RefSeq 106–104, Cabili *et al.*
Analyzed high-throughput experiments	Datasets	322	153
	Conditions	150	67
	Publications	79	22
Analysis of AGO-CLIP-Seq experiments	Framework	microCLIP CLIP-Seq guided model	Intersection of AGO clusters with MREs derived from an *in silico* target prediction model
Experimental Methods	Description of major classes	Reporter genes, northern blot, qPCR, **RIP-qPCR**, biotin miRNA tagging, CLIP-Seq, **CLEAR-CLIP**, **CLIP-chimeric**, **miR-CLIP**, AGO-IP, RNA-Seq, microarrays	Reporter genes, northern blot, qPCR, biotin miRNA tagging, CLIP-Seq, AGO-IP, RNA-Seq, microarrays
LncRNA expression information	Datasets (cell)	48	38
	Datasets (nucleus/cytoplasm)	55	-
Interface	Data visualization, content mining and database inter-connection options	**Re-designed interface**, support of specific queries, **browsing results by different cell type/tissue combinations**, search by location, enhanced filtering options - **transcript biotype, miRNA confidence level, short variant information on MREs -, customizable sorting of results, statistics**, detailed meta-data, **a dedicated module for lncRNA expression profiles in cell and in different cellular compartments**, interconnection with DIANA-tools, UCSC graphical support, **RNAcentral integration, advanced visualizations**	Support of specific queries, search by location, enhanced filtering options including cell type, tissue, species and method, detailed meta-data, cell-type/tissue specific indication of lncRNA expression, interconnection with DIANA-tools, UCSC graphical support

Statistics regarding the total entries, miRNA–lncRNA interactions derived from low-/high-throughput methodologies, the number of miRNAs targeting lncRNA transcripts, the number of lncRNAs harboring MREs, distinct cell types/tissues, curated publications and the incorporated lncRNA resources are provided. The number of analyzed (i) high-throughput datasets and unique studied conditions, (ii) datasets to infer lncRNA expression profiles, is reported. The utilized framework for the analysis of AGO-CLIP-Seq data is mentioned. The incorporated experimental techniques, as well as interface improvements, are displayed and marked as bold in case they constitute additions to LncBase v3.0.

### miRNA and lncRNA sequences

miRNAs were retrieved from miRBase 22 (29). LncRNA transcripts were derived from GENCODE 30 (30). GENCODE is a reference consortium with the most comprehensive annotation of non-coding transcripts. LncRNAs are classified into different categories according to their genomic locus of origin to coding genes. The main categories are the sense intronic, sense overlapping, antisense and intergenic, while the latest GENCODE version also integrates bidirectional promoter and macro lncRNA, as novel lncRNA types. Transcripts annotated as processed transcripts and 3′ prime overlapping ncRNAs are also specified as lncRNAs and were incorporated in our reference annotation. Pseudogenes are included in LncBase v3.0, in consistency with several studies that confirm their interplay with miRNAs (31,32).

According to the latest publication of GENCODE (30), a dedicated effort was made to improve the annotation of coding and non-coding genes. Of note, we observed a significant discrepancy on lncRNA identifiers and on their sequences compared to our previous version, i.e. approximately a 20% and 50% increase was noticed on human and mouse lncRNAs respectively, in the aforementioned lncRNA categories. Our finalized lncRNA collection also incorporates lncRNAs from RefSeq (33) and the publication of Cabili *et al.* (34), that display <90% sequence similarity with GENCODE transcripts, as described in the previous version of our database (25).

The final set is composed of 53 250 and 27 009 lncRNAs and pseudogene transcripts respectively. Specifically, 2297 sense, 11 525 antisense, 19 757 long non-coding intergenic (lincRNAs), 3543 processed transcripts, 309 bidirectional promoter lncRNAs, 38 3′ prime overlapping ncRNAs, 1 macro lncRNA and 14 652 pseudogenes for *Homo sapiens*. The respective set for *Mus musculus* comprises 532 sense, 4361 antisense, 8413 lincRNAs, 2181 processed transcripts, 287 bidirectional promoter lncRNAs, four 3′ prime overlapping ncRNAs, 2 macro lncRNA transcripts and 12 357 pseudogenes.

## METHODS AND RESULTS

### Collection of data

miRNA–lncRNA interactions have been retrieved from manual curation of 159 publications and the analysis of >300 high-throughput datasets. An auxiliary in-house developed text mining pipeline with full-text capacity has been utilized to retrieve publications comprising miRNA–lncRNA pairs and terms conveying ceRNA activity. Sorting of publications was performed based on miRNA target existence. Sentences possibly containing miRNA–lncRNA associations were retained for manual curation.

miRNA–lncRNA interactions supported by high-throughput methodologies were extracted from relevant publications and the analysis of raw libraries. Raw datasets were retrieved from publicly available repositories such as Gene Expression Omnibus (GEO) (35), Encyclopedia of DNA Elements (ENCODE) (36,37) and DNA Data Bank of Japan (DDBJ) (38).

### Analysis of high-throughput data

#### Analysis of AGO-CLIP-Seq data

Raw AGO-CLIP-Seq libraries were quality checked using FastQC (www.bioinformatics.babraham.ac.uk/projects/fastqc/), while adapters/contaminants were detected utilizing in-house developed algorithms and the Kraken suite (39). Upon pre-processing (40), CLIP-Seq libraries were aligned against the reference genomes, i.e. GRCh38 and mm10 assemblies for human and mouse respectively, with GMAP/GSNAP (41) spliced aligner. microCLIP (23) CLIP-Seq-guided algorithm was utilized to identify binding events for the expressed miRNAs. microCLIP is an innovative framework, able to analyze all AGO-enriched regions and to define functional miRNA binding events. In datasets with more than one biological replicates, a miRNA binding event had to be present in at least two replicates (25). Top expressed miRNAs were retrieved either from the relevant publications or from analysis of small RNA-Seq libraries applied to relevant cell types/tissues.

88 AGO-PAR-CLIP and 148 AGO-HITS-CLIP libraries have been analyzed with microCLIP to define ∼370 000 miRNA binding events, corresponding to 37 cell types, 14 tissues and 90 experimental conditions. The sophisticated classification scheme, adopted in microCLIP, maximizes the contribution of a set of 131 descriptors, including AGO-CLIP-related features, such as substitution ratios and coverage metrics, along with characteristics decisive in miRNA–target detection such as the binding type, flanking AU content, energy-related metrics, miRNA-MRE hybrid and sequence-based characteristics. The analysis revealed ∼25 000 lncRNA transcripts with at least one miRNA interaction site. A significant portion of miRNA recognition elements (MREs) was identified on intronic regions, partially attributed to the uncertain splicing of lncRNA transcripts. Therefore, ∼90 000 intronic miRNA binding events are appropriately labeled and provided to users.

The database also indexes 2220 viral miRNA binding events on host lncRNA transcripts, retrieved from the analysis of 16 virus-infected AGO-PAR-CLIP libraries. Expressed viral miRNAs from Epstein-Barr virus (EBV) and Kaposi's sarcoma-associated herpesvirus (KSHV) are associated with ∼2200 miRNA–lncRNA unique interactions.

#### Analysis of miRNA perturbation experiments

For the analysis of microarray miRNA perturbation experiments, we adopted procedures from Mercer *et al.* and Liao *et al.* (42,43) to create custom CDF files for Affymetrix chips Human Genome U133A 2.0, Human Genome U133 Plus 2.0, Human Gene 1.0 ST, Human Exon 1.0 ST, Mouse Genome 430 2.0 and Mouse Gene 1.0 ST. Briefly, probes were aligned to the GRCh38 and mm10 assemblies with Bowtie (44), allowing zero mismatches and multi-maps. HTSeq-count (45) was used to find protein-coding/non-coding genes that each overlap with ≥3 aligned probe sequences. Probes that presented partial overlap with a gene or that overlapped with more than one gene were excluded. Probe set summarization packages were built with makecdfenv package. RMA from packages affy (46) or oligo (47) was employed for probe set summarization and normalization. In cases with replicates, differential expression analysis was performed using moderated *t*-statistics and FDR correction by limma (48). A threshold of 1.5-fold change (FDR < 0.05 where applicable) and the existence of at least one putative canonical binding site for the perturbed miRNA were used as filters to retrieve positive interactions.

In total, 86 microarray perturbation experiments were analyzed, corresponding to 70 cell types and tissues. This process enabled the formation of 1740 and 415 positive miRNA–lncRNA pairs for human and mouse species respectively.

### Tissue/cell type lncRNA expression

Raw RNA-Seq datasets were retrieved from ENCODE (36,37) and GEO (35) repositories, corresponding to 34 distinct cell types and tissues for human and mouse species. RNA-Seq data corresponding to similar cell types and tissues with AGO-CLIP-Seq samples were preferentially selected. Raw datasets were quality checked and pre-processed using FastQC (www.bioinformatics.babraham.ac.uk/projects/fastqc/) and Cutadapt (40). Quantification was conducted at the transcript level, using Salmon (49) version 0.14.1 on quasi-mapping mode and Transcripts Per Million (TPM) values were extracted. 48 whole transcriptome libraries, corresponding to 22 cell types/tissues were analyzed. Transcripts with TPM > 1 were retained, while median TPM values were estimated in case of more than one biological replicates. For the characterization of the subcellular localization of transcripts, 55 libraries from RNA-Seq experiments, conducted separately in nucleus and cytoplasm in 15 distinct cell types/tissues, were pre-processed. Transcripts were filtered out to present TPM > 1 in at least one of the two subcellular compartments. We adopted the Relative Concentration Index (RCI) (50), estimated by transforming the cytoplasmic-to-nuclear TPM fraction into log_2_ scale, to define the trend of lncRNA transcripts localization towards the two different cellular compartments. Human transcriptomes were compiled from ENSEMBL 96 (51), RefSeq 109 (33) and Cabili *et al.* (34), as well as mouse transcriptomes derived from ENSEMBL 96 (51) and RefSeq 106 (33). Details concerning the analyzed RNA-Seq samples are provided in Supplementary Table S1.

### Annotating known variants on MREs

LncBase v3.0 integrates short variant information on experimentally supported miRNA binding events on non-coding transcripts. Variants located on MREs may induce both disruption of these sites and loss of the respective miRNA–lncRNA interactions. Binding sites retrieved from AGO-CLIP-Seq experiments and miRNA–lncRNA chimeric fragments, were intersected with (i) ∼37 million common variations from dbSNP build 151 (26), (ii) 498 490 variants with clinical annotation from ClinVar (27) and (iii) ∼26 million somatic mutations from COSMIC v90 (28). The 67 966 MREs, annotated as variant-related, are located on 9940 human lncRNA transcripts and were associated with 34 438 unique variants, composing a set of 137 882 variant-MRE pairs. Specifically, 48.6% (67 055) of those pairs are associated with common variants, 47.5% (65 476) with somatic mutations and 3.9% (5351) with ClinVar variants.

### Database interface development

#### Database statistics

DIANA-LncBase v3.0 indexes approximately half a million entries, corresponding to the largest collection of experimentally supported cell type and tissue specific miRNA–lncRNA interactions. The incorporated interactions are defined by 14 distinct low-yield and high-throughput methodologies, corresponding to 192 cell types, 52 tissues and 162 experimental conditions. >730 miRNA–lncRNA entries were manually curated while 2094 interactions were extracted from the re-analysis of miRNA-specific transfection/knockdown microarray experiments.

The largest part of the database content is attributed to AGO-CLIP-derived miRNA-binding events. LncBase v3.0 incorporates 2924 miRNA–lncRNA chimeric fragments, while >235 000 interactions have been retrieved from the re-analysis of 236 AGO-CLIP-Seq datasets with a robust CLIP-Seq-guided algorithm.

**Figure 1.**
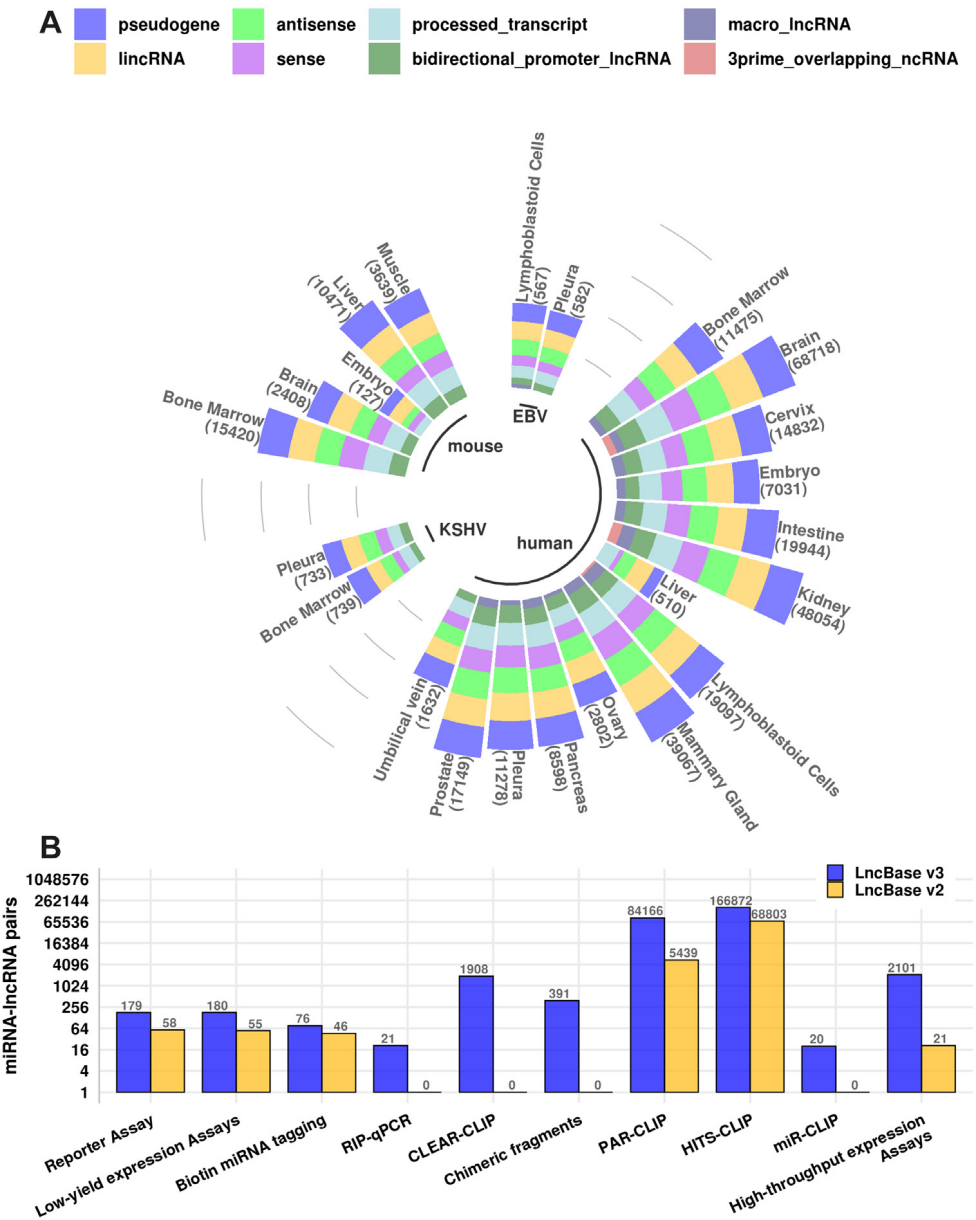
LncBase miRNA–lncRNA pairs. Values are plotted in log_2_ scale. (**A**) miRNA–lncRNA interactions derived from direct high-throughput techniques per tissue and miRNA species. 85 ± 10% of interactions is spatially classified into sense, antisense, lincRNAs and pseudogenes. (**B**) Comparison of LncBase v3.0 and LncBase v2.0 experimentally supported interactions.

The number of miRNA–lncRNA interactions per tissue and miRNA species, retrieved from direct high-throughput techniques, accompanied by the distribution of interactions in the different lncRNA categories, is depicted in Figure 1A. 85 ± 10% of miRNA targets is classified into the main lncRNAs categories (sense, antisense, intergenic) and to pseudogenes. Updated miRNA–lncRNA pairs derived from the different methodologies are summarized in Figure 1B.

#### Interface

In the advanced relational schema of the database, new indices were created in PostgreSQL to ensure quick query execution. A new backend was developed using Java Spring framework and .NET Core 2.2. The database interface has been also redesigned using Angular v.8 and enhanced to provide an intuitive user-friendly application.

#### Querying the database

DIANA-LncBase v3.0 interface comprises two modules. A primary module presenting the experimentally supported interactions and a subsequent module for lncRNA expression profiles. The two modules are inter-connected to easily direct users querying interactions to inspect the expression of lncRNAs under study and vice versa.

##### Module for experimentally supported interactions

**Figure 2.**
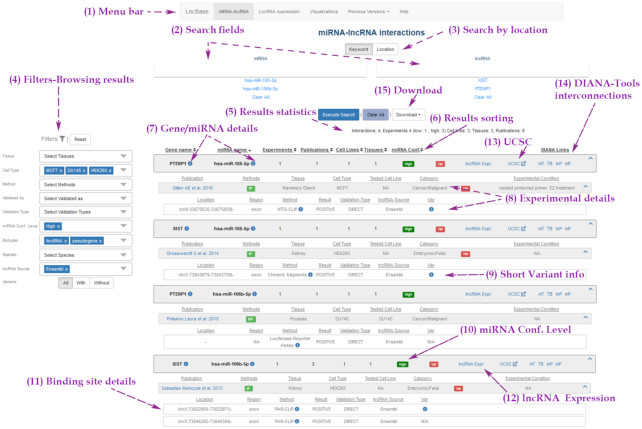
Snapshot depicting the DIANA-LncBase v3.0 interface. Users can explore different database modules and interactive visualizations through a dedicated menu bar (1). They can retrieve interactions by querying with miRNA and/or gene names (2), genomic location (3), and/or by applying different filtering combinations (4). Interactions can be refined with a series of filtering options including cell type/tissue, experimental methodology, transcript category, species and lncRNA annotation source (4). Result statistics are promptly calculated (5). Interactions can be also sorted in ascending or descending order (6). Gene/miRNA details are complemented with active links to Ensembl, RefSeq, miRBase and RNAcentral (7). Additional details regarding the experimental procedures (8), variant information, where applicable (9), as well as miRNA confidence level indication are provided (10). Interactions are accompanied by miRNA-binding site details (11). Inter-connection with the lncRNA expression module is provided (12). miRNA binding events and MRE-overlapping variant genomic locations can be visualized in an interactive UCSC genome browser (13). Links to other DIANA-Tools are also available (14). Users can easily retrieve query results through a dedicated ‘Download’ button (15).

Users can retrieve interactions by (i) performing queries with miRNA and/or gene names - identifiers from ENSEMBL (51), miRBase (29), RefSeq (33) and Cabili *et al.* (34) study are also supported, (ii) applying different combinations of the filtering criteria including species, cell types/tissues and methodologies, (iii) searching a specific genomic location on lncRNA transcripts for the presence of MREs. Filtering options have been also enhanced to offering to users an array of possible filtering combinations (Figure 2). miRNA confidence level indication, incorporated by the latest version of miRBase, and known short variant information on MRE regions are new features that complement the retrieved results and also serve as filters. Helpful variant meta-information, i.e. external identifier, alleles, the exact variant position and links to the original source, is provided where applicable. miRNA binding events per miRNA–lncRNA interaction, coupled with the MRE-overlapping variant genomic locations, can subsequently be visualized in an interactive UCSC genome browser (52) graphic, where users can exploit all the browser options provided by the UCSC team and resources that are integrated there.

##### Module of lncRNA expression profiles

**Figure 3.**
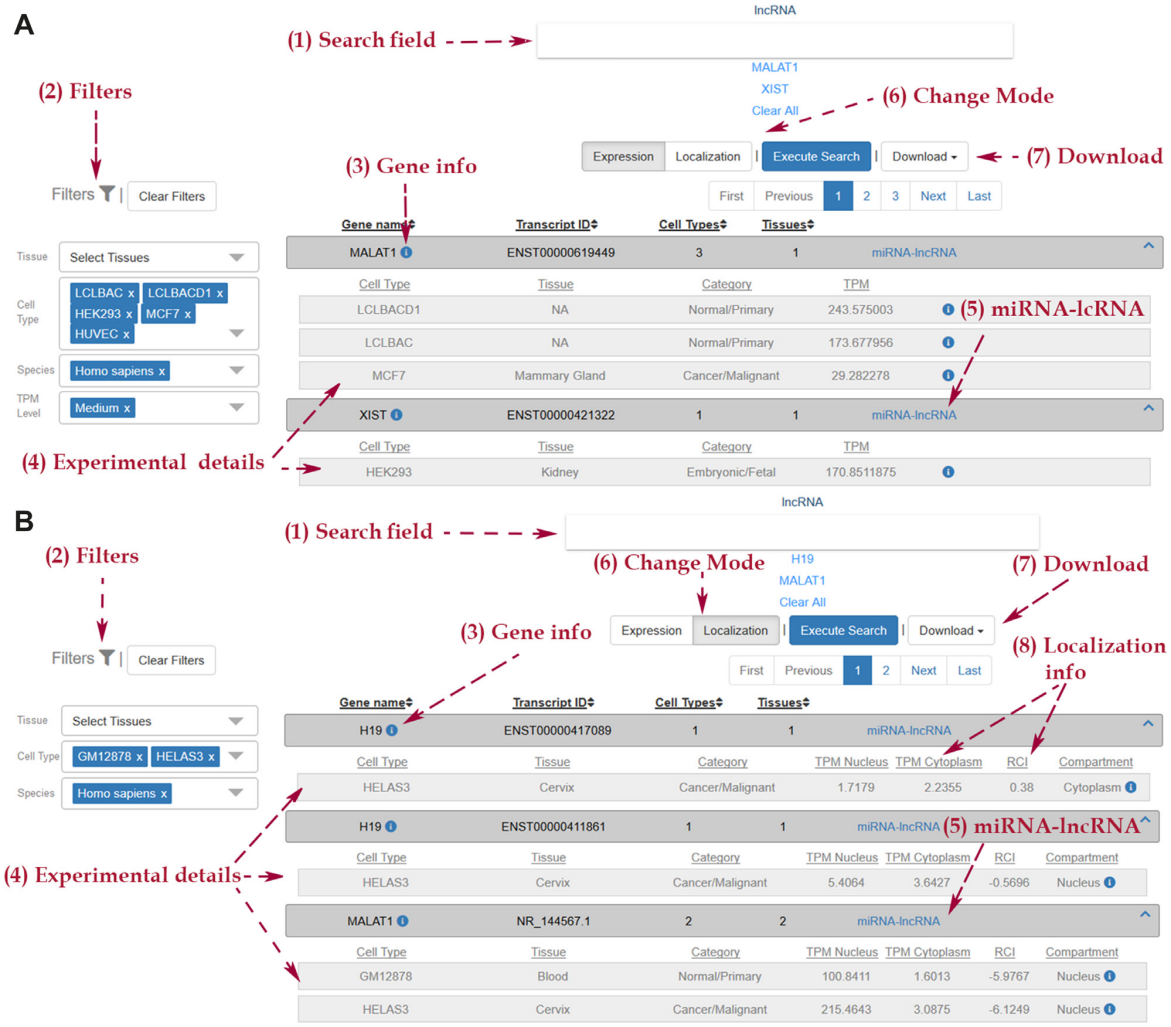
Snapshot depicting the interface of lncRNA expression profile dedicated page. Users can retrieve expression profiles of lncRNAs within the cell (**A**) and comparatively between the nuclear and cytoplasmic subcellular compartments (**B**). They can explore lncRNA abundance by performing queries with gene and/or transcript names (A-1, B-1), as well as combinations of tissues and cell types (A-2, B-2). Gene details, complemented with active links to Ensembl and RefSeq are provided (A-3, B-3). TPM values describing the expression of lncRNAs, accompanied with experimental details are catered to users (A-4). Links directing to the experimentally supported targets module are provided (A-5, B-5). In ‘Localization’ mode, expression TPM values, are provided separately in nucleus and cytoplasm and followed by the RCI value (B-4). Apparent inclination of the sub-localization of lncRNAs is indicated (B-8). Users can easily swap between ‘Localization’ and ‘Expression’ modes and retrieve lncRNA abundance without performing new queries (A-6, B-6). Easy retrieval of query results is also provided through a dedicated ‘Download’ button (A-7, B-7).

LncRNAs expression can be explored either via an inter-connected link in the module of experimentally supported interactions or by applying a query with one or more ncRNA transcripts in the dedicated result page of lncRNA expression. Users can retrieve the expression profiles of lncRNAs (i) within the cell (‘Expression’ mode) and (ii) comparatively between the nuclear and cytoplasmic subcellular compartments (‘Localization’ mode), along a wide range of cell types in human and mouse species. TPM values describing the expression of lncRNAs are provided to users. In case of more than one biological replicates the median TPM value is specified. Specifically, in the ‘Expression’ mode the user can also retrieve results by selecting a particular range of TPM values, described as ‘Low’ (range:1–10), ‘Medium’ (range: 11–600) and ‘High’ (range: >600). In ‘Localization’ mode TPM values, estimated separately in nucleus and cytoplasm, are provided, followed by the RCI value and the apparent inclination of the sub-localization of lncRNAs, either towards the nucleus or the cytoplasm. The user can also retrieve the targets of the specified lncRNAs via a dedicated inter-connected link with the module for the experimentally supported targets (Figure 3).

#### Advanced visualizations

**Figure 4.**
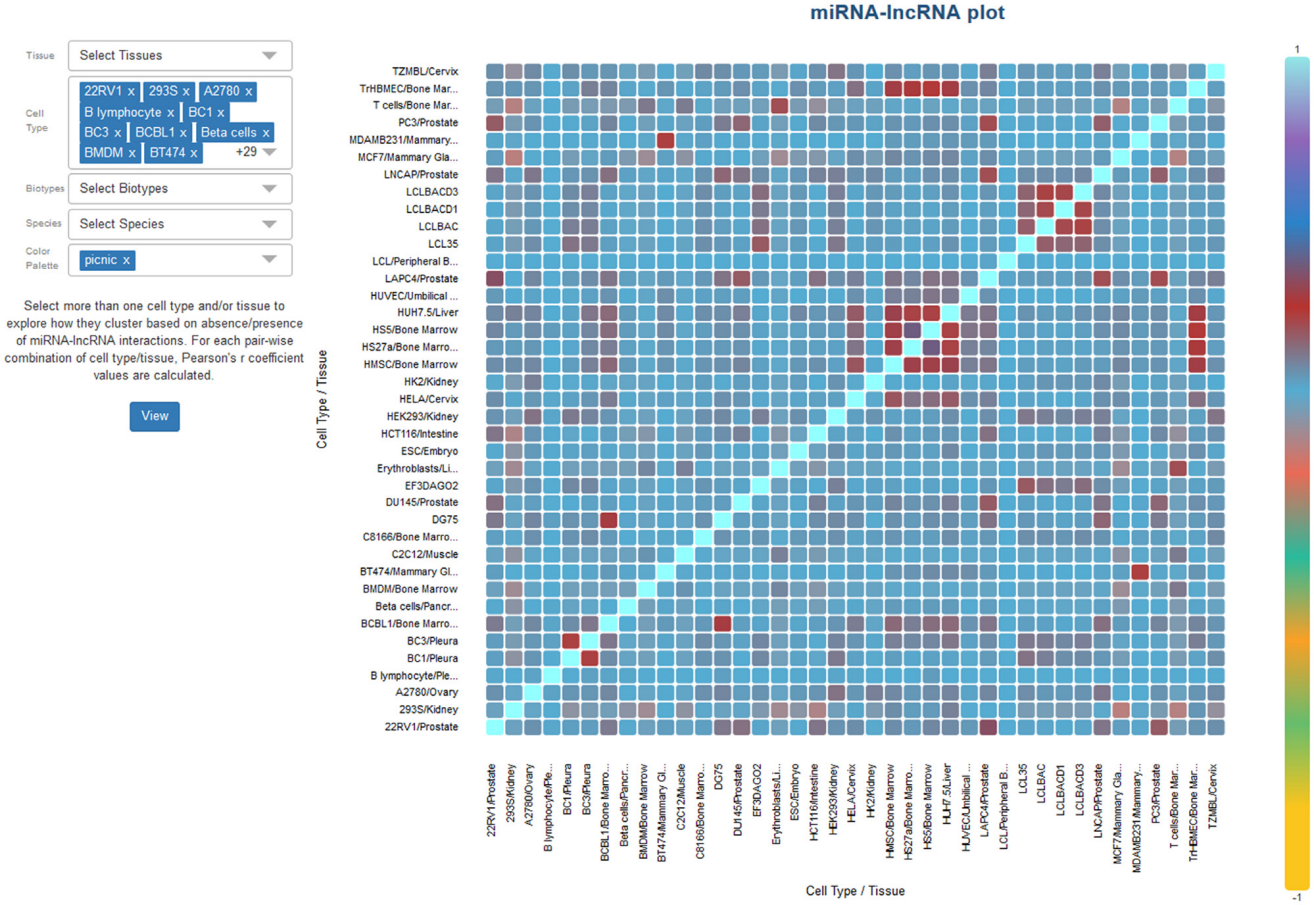
Screenshot depicting DIANA-LncBase v3.0 interactive correlation plot. Users may select more than one cell type and/or tissue to explore their relationship based on absence/presence of AGO-CLIP-Seq derived miRNA–lncRNA interactions. For each pair-wise combination, Pearson's r coefficient values are calculated. The current plot depicts correlations between all human cell types that present AGO-CLIP-Seq derived interactions in LncBase v3.0. Higher correlations are observed among some cell types such as lymphoblastoid, bone marrow-derived and mammary gland cell lines.

**Figure 5.**
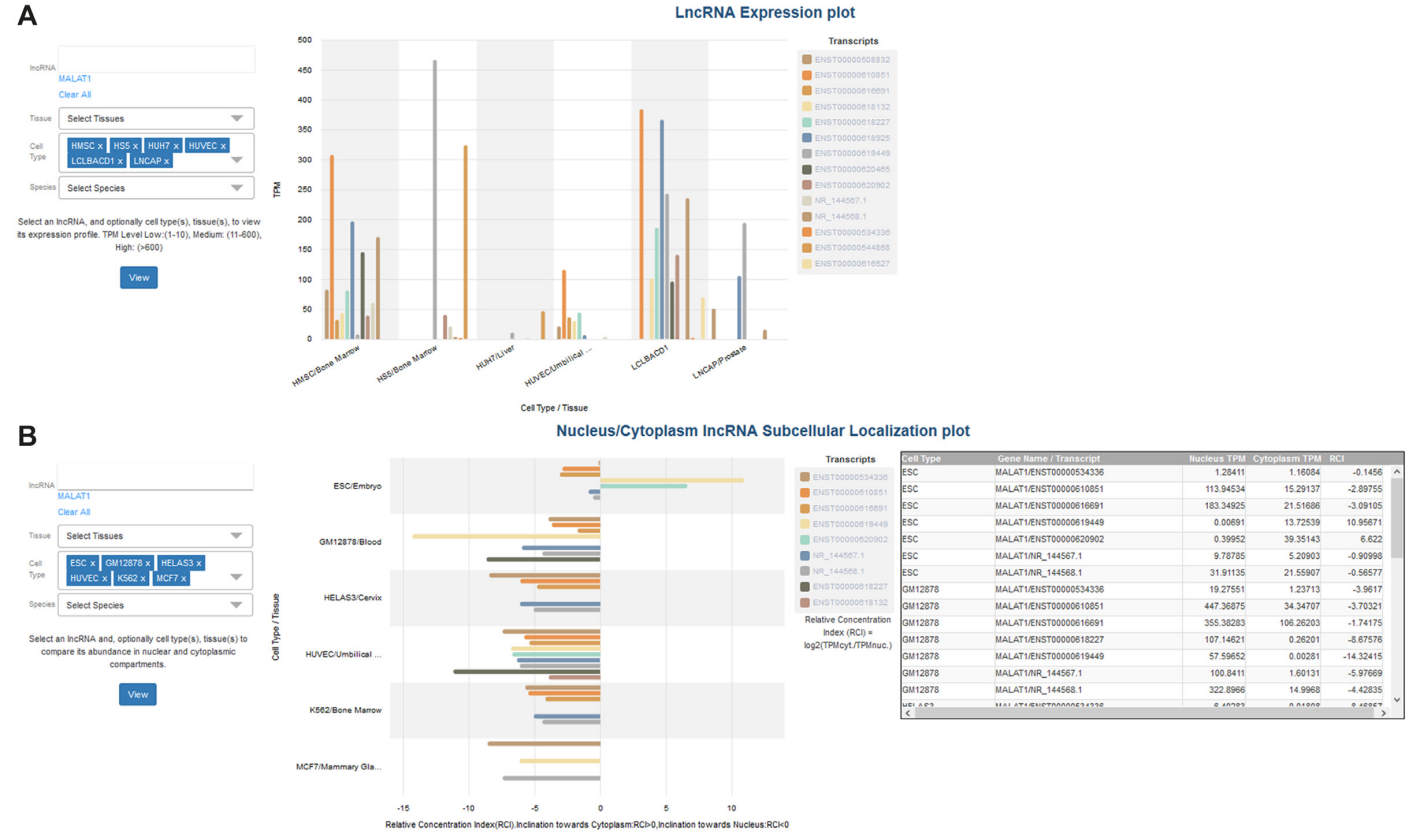
Screenshot depicting DIANA-LncBase v3.0 interactive bar-plots. The user can select an lncRNA, and optionally cell type(s), tissue(s), to view its expression profiles (**A**) and its abundance in nuclear cytoplasmic compartments (**B**). The current bar-plots portray MALAT1 expression. TPM values and RCI values are plotted in ‘LncRNA Expression’ plot (A) and ‘Nucleus/Cytoplasm lncRNA sub-localization’ plot (B), respectively. Negative and positive RCI values denote inclination of the lncRNA expression towards the nucleus and cytoplasm respectively. Users are able to couple RCI values with the nuclear/cytoplasmic lncRNA expression profiles through an inter-connected interactive table (B).

DIANA-LncBase v3.0 also provides interactive visualization plots, implemented using the D3.js JavaScript library. The user can explore a possible clustering of miRNA–lncRNA interactions, retrieved from CLIP-Seq methodologies, in different cell types and tissues via an interactive correlation plot (Figure 4). Interactive bar-plots portraying the expression profiles of lncRNAs within the cell and/or in different subcellular compartments, among distinct cell types, are also provided (Figure 5).

#### Database inter-connections

Since 2015, DIANA-LncBase is integrated in RNAcentral (53). Interactions per miRNA can be viewed in a dedicated page provided by the repository. LncBase v3.0 is also seamlessly inter-connected with the content of other DIANA-tools, including ∼1 million experimentally supported miRNA–mRNA pairs from TarBase (12) and *in silico* identified miRNA targets from microT-CDS, provided as an asset to further explore the activity of miRNA:lncRNA:mRNA endogenous interactions. Predicted miRNA–lncRNA pairs are also supported by LncBase v2.0 (25), while the functionality of miRNAs can be scrutinized through the inter-connection with miRPath (54).

## CONCLUSION

LncRNAs have gradually become a research hotspot, as they seem to participate in several physiological and pathological processes. Their interplay with miRNAs and their prominent role in competing endogenous interactions, in tissue and disease specific contexts, created a constantly evolving research field. The indexing of miRNA–lncRNA interactions was introduced the past few years. LncBase v3.0 showcases the incremental improvement since the previous version by indexing ∼240,000 tissue specific and experimentally supported miRNA–lncRNA interactions. The employment of microCLIP algorithm for the analysis of AGO-CLIP-Seq data provides highly confident miRNA binding events. LncRNA expression profiles accompanied in several cases with their subcellular localization information may serve as a key to further understand lncRNA functions. LncBase v3.0 constitutes an important asset to the research community opening up new possibilities to understand the ncRNA regulatory functions.

## Supplementary Material

gkz1036_Supplemental_File
