# Supplementary material for: DIANA-LncBase v3: indexing experimentally supported miRNA targets on non-coding transcripts
Source: Nucleic Acids Res. 2019 Nov 16;48(D1):D101–10. doi: 10.1093/nar/gkz1036 (PMC7145509; doi:10.1093/nar/gkz1036)
Supplement: gkz1036_Supplemental_File [file gkz1036_supplemental_file.pdf]

Accession	Repository	Cell type/ Tissue	Sub-cellular fraction	Species	Total reads	Sequencing info
**ENCSR000CTL**	encodeproject.org	A549	Cytoplasm	*Homo sapiens*	277M	polyA, PE, 101bp
**ENCSR000CTL**	encodeproject.org	A549	Cytoplasm	*Homo sapiens*	308M	polyA, PE, 101bp
**ENCSR000COR**	encodeproject.org	GM12878	Cytoplasm	*Homo sapiens*	255M	polyA, PE, 76bp
**ENCSR000COR**	encodeproject.org	GM12878	Cytoplasm	*Homo sapiens*	191M	polyA, PE, 76bp
**ENCSR000COV**	encodeproject.org	H1HESC	Cytoplasm	*Homo sapiens*	194M	polyA, PE, 76bp
**ENCSR000CPP**	encodeproject.org	HELAS3	Cytoplasm	*Homo sapiens*	242M	polyA, PE, 76bp
**ENCSR000CPP**	encodeproject.org	HELAS3	Cytoplasm	*Homo sapiens*	208M	polyA, PE, 76bp
**ENCSR000CPF**	encodeproject.org	HEPG2	Cytoplasm	*Homo sapiens*	222M	polyA, PE, 76bp
**ENCSR000CPF**	encodeproject.org	HEPG2	Cytoplasm	*Homo sapiens*	224M	polyA, PE, 76bp
**ENCSR166QLP**	encodeproject.org	HT1080	Cytoplasm	*Homo sapiens*	193M	polyA, PE, 101bp
**ENCSR166QLP**	encodeproject.org	HT1080	Cytoplasm	*Homo sapiens*	195M	polyA, PE, 101bp
**ENCSR000CPA**	encodeproject.org	HUVEC	Cytoplasm	*Homo sapiens*	232M	polyA, PE, 76bp
**ENCSR000CPA**	encodeproject.org	HUVEC	Cytoplasm	*Homo sapiens*	228M	polyA, PE, 76bp
**ENCSR000CTN**	encodeproject.org	IMR90	Cytoplasm	*Homo sapiens*	228M	polyA, PE, 101bp
**ENCSR000CTN**	encodeproject.org	IMR90	Cytoplasm	*Homo sapiens*	288M	polyA, PE, 101bp
**ENCSR000CTU**	encodeproject.org	MCF7	Cytoplasm	*Homo sapiens*	330M	polyA, PE, 101bp
**ENCSR000CTU**	encodeproject.org	MCF7	Cytoplasm	*Homo sapiens*	315M	polyA, PE, 101bp
**ENCSR586SEE**	encodeproject.org	NCIH460	Cytoplasm	*Homo sapiens*	138M	polyA, PE, 101bp
**ENCSR000CPK**	encodeproject.org	NHEK	Cytoplasm	*Homo sapiens*	217M	polyA, PE, 76bp
**ENCSR000CPK**	encodeproject.org	NHEK	Cytoplasm	*Homo sapiens*	196M	polyA, PE, 76bp
**ENCSR291DJH**	encodeproject.org	SKMEL5	Cytoplasm	*Homo sapiens*	173M	polyA, PE, 101bp
**ENCSR291DJH**	encodeproject.org	SKMEL5	Cytoplasm	*Homo sapiens*	190M	polyA, PE, 101bp
**ENCSR569JKX**	encodeproject.org	SKNDZ	Cytoplasm	*Homo sapiens*	150M	polyA, PE, 101bp
**ENCSR569JKX**	encodeproject.org	SKNDZ	Cytoplasm	*Homo sapiens*	178M	polyA, PE, 101bp
**ENCSR000CTR**	encodeproject.org	SKNSH	Cytoplasm	*Homo sapiens*	482M	polyA, PE, 101bp
**ENCSR000CTR**	encodeproject.org	SKNSH	Cytoplasm	*Homo sapiens*	462M	polyA, PE, 101bp
**ENCSR000COK**	encodeproject.org	K562	Cytoplasm	*Homo sapiens*	250M	polyA, PE, 76bp
**ENCSR000COK**	encodeproject.org	K562	Cytoplasm	*Homo sapiens*	177M	polyA, PE, 76bp
**ENCSR000CTM**	encodeproject.org	A549	Nucleus	*Homo sapiens*	369M	polyA, PE, 101bp
**ENCSR000CTM**	encodeproject.org	A549	Nucleus	*Homo sapiens*	204M	polyA, PE, 101bp
**ENCSR000CPO**	encodeproject.org	GM12878	Nucleus	*Homo sapiens*	257M	polyA, PE, 76bp
**ENCSR000CPO**	encodeproject.org	GM12878	Nucleus	*Homo sapiens*	233M	polyA, PE, 76bp
**ENCSR000COW**	encodeproject.org	H1HESC	Nucleus	*Homo sapiens*	208M	polyA, PE, 76bp
**ENCSR000CPQ**	encodeproject.org	HELAS3	Nucleus	*Homo sapiens*	210M	polyA, PE, 76bp
**ENCSR000CPQ**	encodeproject.org	HELAS3	Nucleus	*Homo sapiens*	153M	polyA, PE, 76bp
**ENCSR000CPC**	encodeproject.org	HEPG2	Nucleus	*Homo sapiens*	172M	polyA, PE, 76bp
**ENCSR000CPC**	encodeproject.org	HEPG2	Nucleus	*Homo sapiens*	205M	polyA, PE, 76bp
**ENCSR067UNX**	encodeproject.org	HT1080	Nucleus	*Homo sapiens*	200M	polyA, PE, 101bp
**ENCSR000CPB**	encodeproject.org	HUVEC	Nucleus	*Homo sapiens*	223M	polyA, PE, 76bp
**ENCSR000CTP**	encodeproject.org	IMR90	Nucleus	*Homo sapiens*	342M	polyA, PE, 101bp
**ENCSR000CTP**	encodeproject.org	IMR90	Nucleus	*Homo sapiens*	335M	polyA, PE, 101bp
**ENCSR000CTO**	encodeproject.org	MCF7	Nucleus	*Homo sapiens*	297M	polyA, PE, 101bp

**ENCSR000CTO**	encodeproject.org	MCF7	Nucleus	*Homo sapiens*	283M	polyA, PE, 101bp
**ENCSR625QJI**	encodeproject.org	NCIH460	Nucleus	*Homo sapiens*	179M	polyA, PE, 101bp
**ENCSR625QJI**	encodeproject.org	NCIH460	Nucleus	*Homo sapiens*	236M	polyA, PE, 101bp
**ENCSR000CPJ**	encodeproject.org	NHEK	Nucleus	*Homo sapiens*	208M	polyA, PE, 76bp
**ENCSR000CPJ**	encodeproject.org	NHEK	Nucleus	*Homo sapiens*	240M	polyA, PE, 76bp
**ENCSR201WVA**	encodeproject.org	SKMEL5	Nucleus	*Homo sapiens*	129M	polyA, PE, 101bp
**ENCSR201WVA**	encodeproject.org	SKMEL5	Nucleus	*Homo sapiens*	154M	polyA, PE, 101bp
**ENCSR255NYQ**	encodeproject.org	SKNDZ	Nucleus	*Homo sapiens*	157M	polyA, PE, 101bp
**ENCSR255NYQ**	encodeproject.org	SKNDZ	Nucleus	*Homo sapiens*	176M	polyA, PE, 101bp
**ENCSR000CTS**	encodeproject.org	SKNSH	Nucleus	*Homo sapiens*	487M	polyA, PE, 101bp
**ENCSR000CTS**	encodeproject.org	SKNSH	Nucleus	*Homo sapiens*	299M	polyA, PE, 101bp
**ENCSR000CPS**	encodeproject.org	K562	Nucleus	*Homo sapiens*	234M	polyA, PE, 76bp
**ENCSR000CPS**	encodeproject.org	K562	Nucleus	*Homo sapiens*	211M	polyA, PE, 76bp
**GSM3619600**	ncbi.nlm.nih.gov/geo	A2780	Cell	*Homo sapiens*	94M	polyA, PE, 150bp
**GSM3619601**	ncbi.nlm.nih.gov/geo	A2780	Cell	*Homo sapiens*	85M	polyA, PE, 150bp
**GSM3309425**	ncbi.nlm.nih.gov/geo	BC1	Cell	*Homo sapiens*	82M	total RNA, PE, 150bp
**GSM3926177**	ncbi.nlm.nih.gov/geo	Beta cells	Cell	*Homo sapiens*	108M	total RNA, PE, 101bp
**GSM3926178**	ncbi.nlm.nih.gov/geo	Beta cells	Cell	*Homo sapiens*	119M	total RNA, PE, 101bp
**GSM3926179**	ncbi.nlm.nih.gov/geo	Beta cells	Cell	*Homo sapiens*	118M	total RNA, PE, 101bp
**ENCSR000AEY**	encodeproject.org	Brain	Cell	*Homo sapiens*	229M	total RNA, PE, 101bp
**ENCSR000AEY**	encodeproject.org	Brain	Cell	*Homo sapiens*	280M	total RNA, PE, 101bp
**GSM3466389**	ncbi.nlm.nih.gov/geo	HEK293	Cell	*Homo sapiens*	100M	polyA, PE, 150bp
**GSM3466390**	ncbi.nlm.nih.gov/geo	HEK293	Cell	*Homo sapiens*	102M	polyA, PE, 150bp
**ENCSR000CPR**	encodeproject.org	HELAS3	Cell	*Homo sapiens*	235M	polyA, PE, 76bp
**ENCSR000CPR**	encodeproject.org	HELAS3	Cell	*Homo sapiens*	242M	polyA, PE, 76bp
**GSM2890251**	ncbi.nlm.nih.gov/geo	H9HESC	Cell	*Homo sapiens*	112M	total RNA, PE, 99bp
**GSM2890252**	ncbi.nlm.nih.gov/geo	H9HESC	Cell	*Homo sapiens*	129M	total RNA, PE, 99bp
**GSM2890253**	ncbi.nlm.nih.gov/geo	H9HESC	Cell	*Homo sapiens*	133M	total RNA, PE, 99bp
**GSM2890254**	ncbi.nlm.nih.gov/geo	H9HESC	Cell	*Homo sapiens*	110M	total RNA, PE, 99bp
**GSM2890255**	ncbi.nlm.nih.gov/geo	H9HESC	Cell	*Homo sapiens*	115M	total RNA, PE, 99bp
**GSM2288692**	ncbi.nlm.nih.gov/geo	HK2	Cell	*Homo sapiens*	45M	polyA, PE, 125bp
**GSM2944155**	ncbi.nlm.nih.gov/geo	HMSC	Cell	*Homo sapiens*	78M	total RNA, PE, 141bp
**GSM2944156**	ncbi.nlm.nih.gov/geo	HMSC	Cell	*Homo sapiens*	74M	total RNA, PE, 142bp

**GSM2693413**	ncbi.nlm.nih.gov/geo	HS5	Cell	*Homo sapiens*	58M	polyA, PE, 101bp
**GSM3717028**	ncbi.nlm.nih.gov/geo	HUH7	Cell	*Homo sapiens*	43M	total RNA, PE, 150bp
**GSM3752526**	ncbi.nlm.nih.gov/geo	HUVEC	Cell	*Homo sapiens*	224M	total RNA, PE, 150bp
**GSM3752527**	ncbi.nlm.nih.gov/geo	HUVEC	Cell	*Homo sapiens*	202M	total RNA, PE, 150bp
**GSM3752528**	ncbi.nlm.nih.gov/geo	HUVEC	Cell	*Homo sapiens*	214M	total RNA, PE, 150bp
**GSM1133248**	ncbi.nlm.nih.gov/geo	LCLBACD1	Cell	*Homo sapiens*	77M	total RNA, PE, 50bp
**GSM1133249**	ncbi.nlm.nih.gov/geo	LCLBACD1	Cell	*Homo sapiens*	74M	total RNA, PE, 50bp
**GSM1133250**	ncbi.nlm.nih.gov/geo	LCLBACD2	Cell	*Homo sapiens*	46M	total RNA, PE, 50bp
**GSM1133251**	ncbi.nlm.nih.gov/geo	LCLBACD3	Cell	*Homo sapiens*	74M	total RNA, PE, 50bp
**GSM1133247**	ncbi.nlm.nih.gov/geo	LCLBAC	Cell	*Homo sapiens*	68M	total RNA, PE, 50bp
**GSM2730123**	ncbi.nlm.nih.gov/geo	Liver	Cell	*Homo sapiens*	62M	total RNA, PE, 50bp
**GSM2730124**	ncbi.nlm.nih.gov/geo	Liver	Cell	*Homo sapiens*	86M	total RNA, PE, 50bp
**GSM2730125**	ncbi.nlm.nih.gov/geo	Liver	Cell	*Homo sapiens*	94M	total RNA, PE, 50bp
**GSM2730126**	ncbi.nlm.nih.gov/geo	Liver	Cell	*Homo sapiens*	88M	total RNA, PE, 50bp
**GSM3294563**	ncbi.nlm.nih.gov/geo	LNCAP	Cell	*Homo sapiens*	98M	total RNA, PE, 51bp
**GSM3294564**	ncbi.nlm.nih.gov/geo	LNCAP	Cell	*Homo sapiens*	86M	total RNA, PE, 51bp
**GSM3294565**	ncbi.nlm.nih.gov/geo	LNCAP	Cell	*Homo sapiens*	99M	total RNA, PE, 51bp
**GSM2072541**	ncbi.nlm.nih.gov/geo	PC3	Cell	*Homo sapiens*	175M	total RNA, PE, 101bp
**GSM2072542**	ncbi.nlm.nih.gov/geo	PC3	Cell	*Homo sapiens*	266M	total RNA, PE, 101bp
**GSM3736829**	ncbi.nlm.nih.gov/geo	MCF7	Cell	*Homo sapiens*	48M	total RNA, PE, 100bp
**ENCSR000BZJ**	encodeproject.org	Brain	Cell	*Mus musculus*	346M	polyA, PE, 101bp
**ENCSR000BZJ**	encodeproject.org	Brain	Cell	*Mus musculus*	341M	polyA, PE, 101bp
**GSM2639637**	ncbi.nlm.nih.gov/geo	C2C12	Cell	*Mus musculus*	135M	total RNA, PE, 75bp
**GSM2639638**	ncbi.nlm.nih.gov/geo	C2C12	Cell	*Mus musculus*	116M	total RNA, PE, 75bp
**GSM2639639**	ncbi.nlm.nih.gov/geo	C2C12	Cell	*Mus musculus*	183M	total RNA, PE, 75bp

**GSM2639640**	ncbi.nlm.nih.gov/geo	C2C12	Cell	*Mus musculus*	162M	total RNA, PE, 75bp
**GSM3602657**	ncbi.nlm.nih.gov/geo	ESC	Cell	*Mus musculus*	82M	total RNA, PE, 76bp
**GSM3602658**	ncbi.nlm.nih.gov/geo	ESC	Cell	*Mus musculus*	83M	total RNA, PE, 76bp

Table S1: Details regarding the analysed RNA-Seq libraries. The datasets were utilized to estimate lncRNA transcript expression profiles in a wide range of cell types/tissues and different cellular compartments (nucleus/cytoplasm).
